# Testing for coronarvirus disease 2019 before cardiac surgery—safe outcome of infected patients

**DOI:** 10.1186/s13019-022-01960-1

**Published:** 2022-08-24

**Authors:** Torbjörn Ivert, Magnus Dalén

**Affiliations:** 1 Department of Cardiothoracic Surgery, Karolinska University Hospital, Eugeniavägen 23, C12:28, 171 76 Stockholm, Sweden; 2 Department of Molecular Medicine and Surgery, Karolinska Institutet, Stockholm, Sweden

**Keywords:** COVID-19, Cardiac surgery, Preoperative PCR testing

## Abstract

**Background:**

The aim was to analyze routine preoperative testing for coronavirus disease 2019 (COVID-19) performed to avoid infected cardiac surgical patients transmitting virus during the pandemic.

**Methods:**

Every patient scheduled to undergo cardiac surgery from March 2020 through December 2021 had preoperative polymerase-chain-reaction (PCR) test for COVID-19 by nasopharynx swabs. Any history of COVID-19 was recorded.

**Results:**

In 15 of 1870 patients (0.8%) with minimal or no airway symptoms unexpected positive PCR tests were detected, and surgery was deferred for two weeks. Totally 38 patients with negative tests had recovered without sequelae from previous COVID-19 a mean of 5 months before the operation. Sixteen patients (0.8%) developed airway symptoms within six weeks after the operation and had positive COVID-19 tests. Body Mass Index was higher and female gender, diabetes mellitus, chronic obstructive pulmonary disease and reduced left ventricular ejection fraction were more common in patients with than in those without COVID-19. Early postoperative outcomes did not differ significantly in patients with versus without COVID-19.

**Conclusions:**

An unexpected preoperative positive COVID-19 test was detected in less than one percent of patients admitted for cardiac surgery during the pandemic. These operations were deferred to avoid transmission of virus in the hospital. Additionally, one percent of patients were diagnosed with positive COVID-19 tests within six weeks after the operation. There was no outbreak of COVID-19 among hospital staff or patients. All patients with COVID-19 before the operation were operated on safely and postoperative outcomes did not differ significantly compared with COVID-19 negative patients.

## Background

The novel coronavirus disease 2019 (COVID-19) was declared pandemic on March 11, 2020 by the World Health Organization. Severe acute respiratory corona virus (SARS-CoV-2) cause an infectious syndrome and in some subjects hypoxic respiratory failure that may require long hospitalization with sometimes fatal outcome [1, 2]. During 2020 through 2021 the number of subjects in Stockholm diagnosed with COVID-19 occurred in waves. A fourth smaller wave occurred in the early fall of 2021 and was followed by increasing number of cases at the end of the year. The acute burden during the pandemic on healthcare limited the resources for surgery [3, 4]. To maintain capacity to perform cardiac surgery and avoid transmission of virus among patients and hospital staff it was necessary to avoid cases admitted with ongoing COVID-19. Patients scheduled for elective heart surgery were asked not to come the hospital in case of airway symptoms. A decision was taken to test for COVID-19 in every patient before the operation.

Our aim was to analyse incidence of COVID-19 among patients admitted for cardiac surgery by preoperative PCR (polymerase chain reaction) tests, to record any history of COVID-19 before or early after the operation and to analyse postoperative outcome.

### Patients and methods

We included all 1870 patients who underwent cardiac surgery operation from March 2020 through December 2021 at the Karolinska University in Stockholm, Sweden. The capacity to perform elective as well as acute open-heart surgery was not reduced during the pandemic as symptomatic COVID-19 cases referred to the hospital were allocated to other wards. Every patient regardless of previous infection were screened for COVID-19 on nasopharynx swabs by real-time reverse transcription PCR test for SARS-CoV-2-RNA (ribonucleic acid) with high sensitivity [5]. The samples were collected within 48 hours before planned surgery by a trained nurse in extensive personal protective equipment. Acute and urgent procedure were performed regardless of test results.

### Definitions

Severe COVID-19 was defined as need for intensive care treatment because of respiratory failure. Hypertension was defined as treatment with an anti-hypertensive drug and hyperlipidaemia as treatment with statins. Chronic obstructive pulmonary disease was defined as medication with bronchodilators or steroids for lung disease. Diabetes included treatment with either insulin, oral hyperglycemic drugs, or diet. The risk of surgery was assessed by using the European System for Cardiac Operative Risk Evaluation Score (EuroSCORE) II [6]

### Statistical methods

Continuous variables are presented with arithmetic mean and one standard deviation (SD). Student’s *t* test was used to compare continuous variables in patients with COVID-19 to those not infected. In case of skew distribution, the Mann–Whitney’s *U* test was used. The chi-square method with Yate’s correction for continuity was applied to analyze proportions for categorical variables. *P *values < 5% were considered statistically significant. The calculations were performed using STATISTICA 13 (Stat Soft, Dell).

## Results

### Preoperative PCR tests

In 15 of the 1870 patients (0.8%) an unexpected positive SARS-CoV-2 PCR test was detected at the preoperative screening. Two of these 15 patients had been previously vaccinated. Five patients reported minimal airway symptoms and 10 were asymptomatic. One 44-year-old male with aortic cusp vegetations, aortic regurgitation and cerebral emboli caused by Staphylococcus aureus endocarditis and a 66-year old nurse who developed massive mitral regurgitation seven years after previous mitral annuloplasty, underwent urgent valve surgery. In 13 patients surgery was postponed 2 weeks, six of whom had non-ST elevation myocardial infarction and needed coronary artery bypass grafting. Except for transient atrial fibrillation in two patients all operations were performed without symptoms of COVID-19 infection, other complications or deaths within 30 days.

### COVID-19 infection before the operation

In addition, totally 38 patients (2.0%) had recovered from symptomatic COVID-19 with a positive PCR test one to 12 months (mean 5 months) before the operation. The PCR test was negative before the operation. Sixteen of the patients (42%) had needed treatment in hospital because of respiratory distress. Five of whom had required a period of mechanical ventilation. All had recovered without sequelae and underwent coronary artery bypass grafting (CABG) (*n *= 14) or valve surgery (*n *= 24) without major complications or deaths within 30 days.

### COVID-19 infection after the operation

Sixteen of the 1870 patients (0.8%) who developed airway symptoms and fever within six weeks after the operation had positive PCR test for COVID-19. They were diagnosed while still in hospital, at a rehabilitation facility or recorded at our routine postoperative out-clinic follow-up of all operated patients. All were initially operated on without complications. Nine patients had onset of symptoms within two weeks after the operation. Nine of the patients had severe symptoms with increased oxygen demand and computed tomography scans of the lungs with changes typical for COVID-19. Two had pulmonary embolism. A 70-year-old woman had replacement of the ascending aorta and died from COVID-related respiratory failure two weeks after the operation. There were many patients with slightly postoperative elevated body temperature who had negative COVID-19 tests.

### Analyses of all COVID-19 cases

**Fig. 1:**
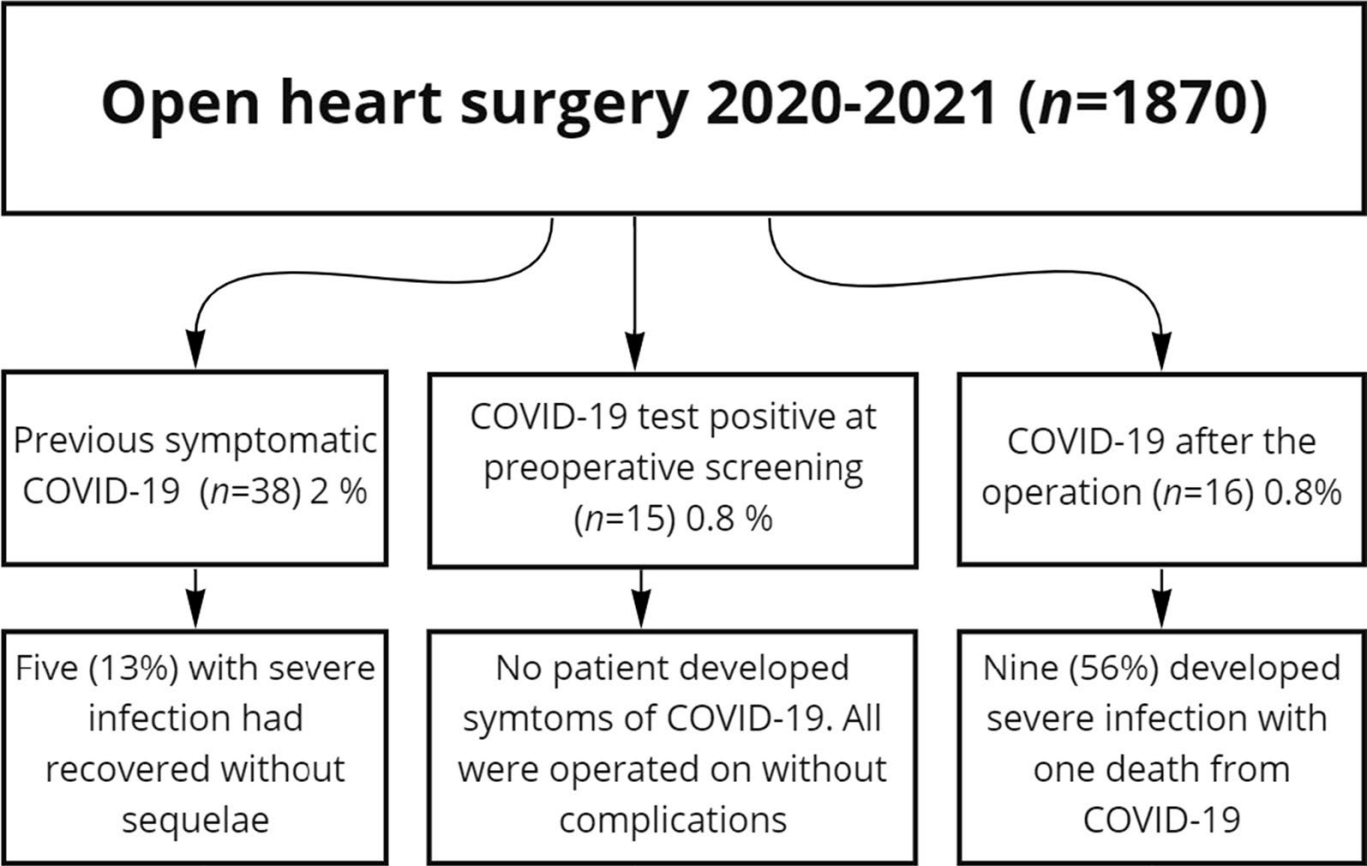
Flowchart showing number cases with a SARS-CoV-2 PCR positive test and severity of COVID-19 before, at screening and after open heart surgery in 1870 patients operated on during 2020 and 2021.

**Fig. 2:**
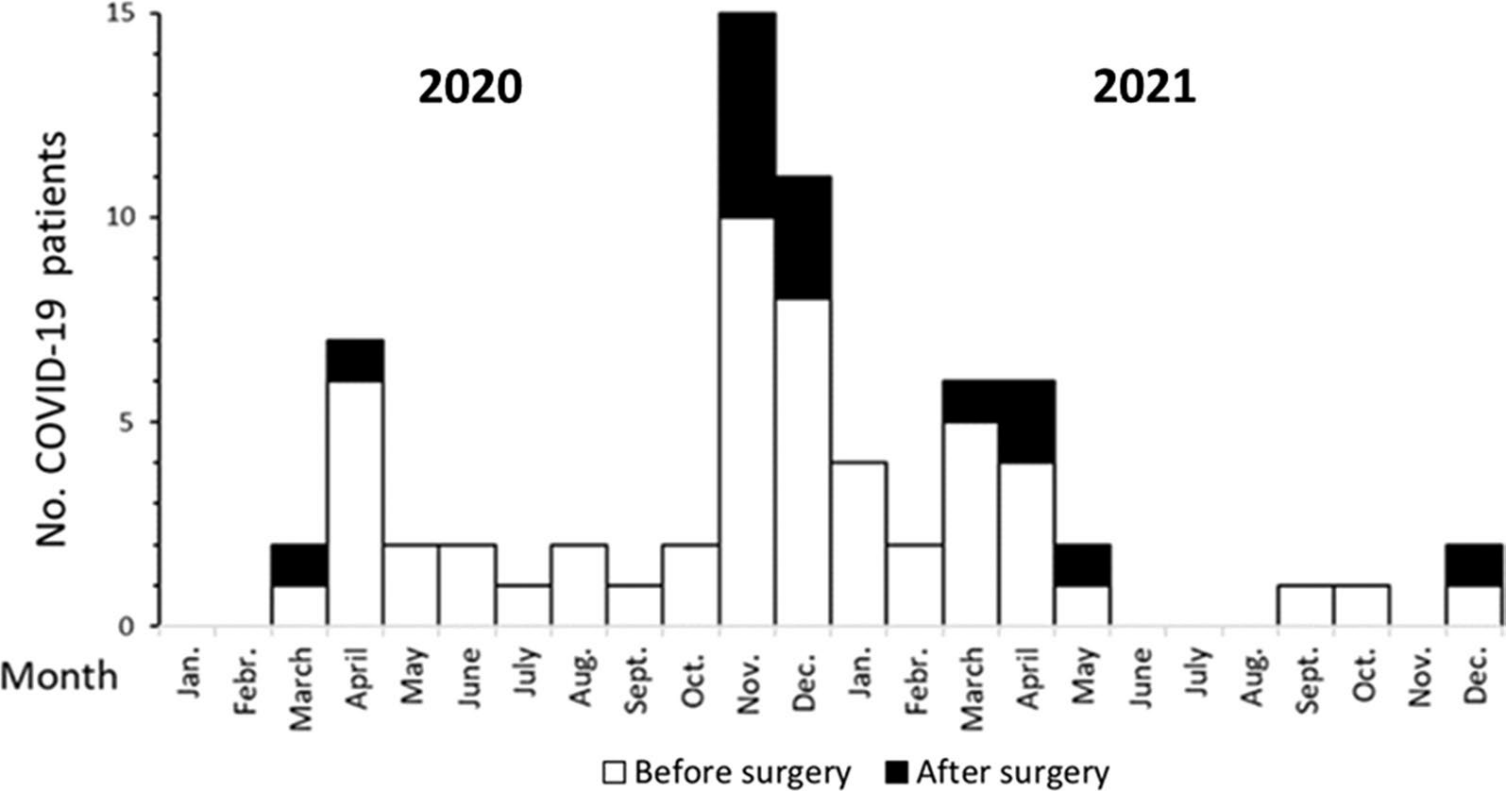
Distribution over time of COVID-19 diagnosis in 69 patients with a SARS-CoV-2 PCR positive test among 1870 patients undergoing open heart surgery during 2020 and 2021.Black bars indicated cases with positive test after the operation.

We thus encountered a total of 69 COVID-19 cases between March 2020 and December 2021 (Fig. 1). Most cases occurred from November 2020 until May 2021 and a few cases were diagnosed during the fall of 2021 (Fig. 2). Body mass index was higher and female gender, diabetes, obstructive pulmonary disease and reduced left ventricular ejection fraction more common in COVID-19 patients than those without COVID-19 (Table 1). Serum creatinine before the operation and EuroSCORE II risk did not differ significantly. Type of surgical procedure performed, mortality within 30 days and complication rates did not differ significantly in the two groups (Table 2).

**Table 1:** Preoperative characteristic in patients with and without COVID-19 who underwent open cardiac surgery from March 2020 through December 2021.

	COVID-19 (n = 69^a^)	Non COVID-19 (n = 1801)	*P* value
Age (years)	62 (10)	62 (13)	0.90
Female gender	27 (39.1)	446 (24.8)	0.01
BMI (kg/m^2^)	28.4 (5)	27.0 (6)	0.04
Creatinine (µmol/L)	79.4 (25.0)	86 (39.1)	0.15
EuroSCORE II	4.0 (6.5)	4.6 (6.9)	0.40
Previous smoking	36 (52.1)	717 (39.8)	0.23
Hypertension	47 (68.1)	1044 (58.0)	0.12
Hyperlipidaemia	39 (56.5)	901 (50.0)	0.35
Diabetes mellitus	20 (29.0)	341 (18.9)	0.05
COPD	12 (17.4)	159 (8.8)	0.01
LVEF ≤ 50%	45 (65.2)	524 (29.0)	< 0.001

BMI, Body Mass Index; COPD, chronic obstructive pulmonary disease; EuroSCORE, European System for Cardiac Operative Risk Evaluation Score II [6]; LVEF, left ventricular ejection fraction.

^a^38 (55%) previous COVID-19, 15 (22%) positive on admission, 16 (23%) postoperative COVID-19. Data presented as numbers with percent or mean with one standard deviation

**Table 2:** Operative data and early postoperative outcome in patients with and without COVID-19 who had cardiac surgery from March 2020 through December 2021.

	COVID-19 (*n *= 69)	Non COVID-19 (*n *= 1801)	*P* value
Urgent operation	28(40.6)	608(33.8)	
Emergent operation	3(4.3)	106(5.9)	
Valve surgery	33(47.8)	756(42.0)	0.19
Isolate CABG	27(39.1)	591(32.8)	0.34
Valve and CABG	1(1.4)	75(4.2)	0.44
Aortic surgery	5(7.2)	286(15.9)	0.76
Other procedures	3(4.3)	93(5.2)	0.98
Mortality ≤ 30 days	1 (0.9)	20 (1.1)	0.77
Re-entry for bleeding	2 (2.9)	84 (4.7)	0.74
Stroke	0	40 (2.2)	0.42
Respirator ≥ 2 days	2 (2.9)	34 (1.9)	0.84
Atrial fibrillation	11 (15.9)	374 (20.8)	0.50
Pleuracentesis	5 (7.2)	70 (3.9)	0.24
Pericardiocentesis	3 (4.3)	31 (1.7)	0.23

Data presented as numbers with percent

CABG, coronary artery bypass grafting

## Discussion

**Fig. 3:**
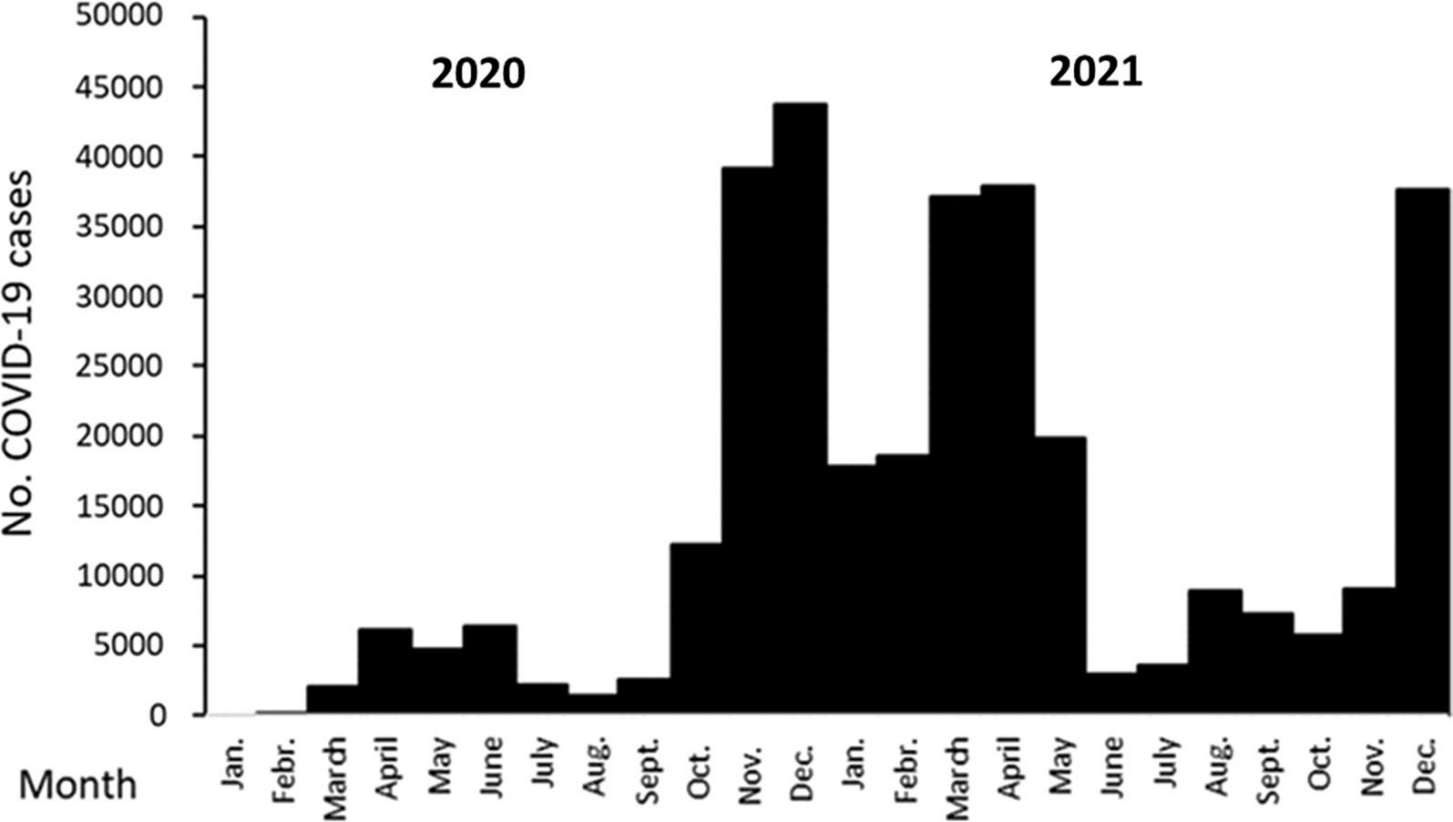
Distribution over time of 326,995 subjects with a SARS-CoV-2 PCR positive test among the population of 975,500 inhabitants (34%) in Stockholm during 2020 and 2021 Official statistics from The Public Health Agency of Sweden (https://www.folkhalsomyndigheten.se).

We had initially assumed that the maximal number of cases with COVID-19 would occur during 2020 but experienced waves of cases with incidence curves similar to that for transmission in the community of entire Stockholm area (Fig. 3). Volume of intensive care beds could be adapted according to demand, and shortness of beds or staff did not restrict capacity to perform cardiac surgery in Stockholm during the pandemic. A general vaccination program was started in Sweden during the early spring of 2021 and may have contributed to fewer cases and less severe symptoms during the fall of 2021.

We used active surveillance by preoperative testing for COVID-19 in every patient as has been recommended by other authors [2]. An unexpected positive COVID-19 test was detected in less than one percent patients admitted for cardiac surgery. During the pandemic all subjects were advised social distancing, restrictive contact with others, if possible to work from home and avoid shopping and public transportation to reduce transmission of virus. An additional one percent developed COVID-19 within six weeks after the operation despite single rooms in the hospital, compulsory use of personal protection device as gloves, mouth guard and face shields by hospital staff during direct patient contact in the hospital wards and that hospital visit from relatives was not allowed.

Patients with previous COVID-19 were operated on without increased incidence of pulmonary or other postoperative complications. Cardiopulmonary bypass was conducted without complications regardless of previous COVID-19.

We recorded a higher percentage of women in COVID-19 patients than in non-COVID-19 patients. In an analysis of data from several European countries middle aged women are more vulnerable to COVID-19 than men but fatality is higher in men [7]. In our small cohort comorbidities as higher body mass index, diabetes, COPD and reduced left ventricular function were more common in COVID-19 patients than in those not infected. This agrees with findings in other reports that main risk factors for COVID-19 are hypertension, diabetes, COPD, and cardiovascular disease [4, 8]. The risk of severe COVID-19 is four times increased in patients with COPD and twice times increased in smokers [9]. Obesity and diabetes are risk factors for severe COVID-19 [10].

## Conclusions

During the pandemic about one percent of patients admitted for cardiac surgery had an unexpected positive PCR test for COVID-19 before the operation despite no or minimal symptoms. Further one percent developed COVID-19 within six weeks after the operation. COVID-19 cases were more often females had higher body mass index, diabetes, obstructive pulmonary disease and reduced left ventricular function than non-COVID-19 cases. There was no outbreak of COVID-19 among staff or patients. Patients with healed COVID-19 were operated on safely with postoperative outcomes not significantly different compared with COVID-19 negative patients.

### Limitations

False negative PCR tests may occur for reasons as sampling error and timing of sample and initially negative tests may become positive with subsequent testing [11]. Repeated tests increase sensitivity to 98% [12]. Furthermore COVID-19 is associated with a wide spectrum of symptoms and clinical signs may be misleading. Our report therefore probably underreports the true number of COVID-19 cases. There might have been patients with subclinical COVID-19 and a false negative test before the operation. Personal protection device was mandatory and there was no major outbreak of the infection in the hospital although isolated individuals were afflicted. Certainly, patients may have acquired asymptomatic COVID-19 after the operation that was not registered.

## Data Availability

Data are used for this study are available from the corresponding author on reasonable request.
